# How can microbial population genomics inform community ecology?

**DOI:** 10.1098/rstb.2019.0253

**Published:** 2020-03-23

**Authors:** David VanInsberghe, Philip Arevalo, Diana Chien, Martin F. Polz

**Affiliations:** 1 Department of Civil and Environmental Engineering, Massachusetts Institute of Technology, Cambridge, MA, USA; 2 Graduate Program in Microbiology, Massachusetts Institute of Technology, Cambridge, MA, USA; 3 Department of Ecology and Evolution, University of Chicago, Chicago, IL, USA; 4 Department of Microbial Ecology, University of Vienna, Vienna, Austria

**Keywords:** populations, species, genetic sweep, microbial ecology, population genomics, gene flow

## Abstract

Populations are fundamental units of ecology and evolution, but can we define them for bacteria and archaea in a biologically meaningful way? Here, we review why population structure is difficult to recognize in microbes and how recent advances in measuring contemporary gene flow allow us to identify clearly delineated populations among collections of closely related genomes. Such structure can arise from preferential gene flow caused by coexistence and genetic similarity, defining populations based on biological mechanisms. We show that such gene flow units are sufficiently genetically isolated for specific adaptations to spread, making them also ecological units that are differentially adapted compared to their closest relatives. We discuss the implications of these observations for measuring bacterial and archaeal diversity in the environment. We show that operational taxonomic units defined by 16S rRNA gene sequencing have woefully poor resolution for ecologically defined populations and propose monophyletic clusters of nearly identical ribosomal protein genes as an alternative measure for population mapping in community ecological studies employing metagenomics. These population-based approaches have the potential to provide much-needed clarity in interpreting the vast microbial diversity in human and environmental microbiomes.

This article is part of the theme issue ‘Conceptual challenges in microbial community ecology’.

## 1. Introduction

Take any introductory biology textbook and you will probably find evolution defined as change in the genetic makeup of populations. Being defined as the locally coexisting representatives of species, populations are, in practice, also the units of diversity that are used when we wish to measure species diversity to assess ecological interactions as well as ecosystem stability and resilience [1]. For microbes, however, populations have been notoriously difficult to define [2], and we use arbitrary units of diversity to measure the genetic makeup of communities [3]. This difficulty in defining populations is, of course, rooted in the absence of a biologically meaningful species concept for bacteria and archaea [3–6]. Without clearly defined populations, many of the most fundamental questions in community ecology are difficult to answer. For example, do disturbances lead to changes in genotypic composition within populations or to species turnover? Differentiating between these possibilities is a meaningful question because shifts in genotype within a population may be far less disruptive to ecological networks than wholesale changes in species composition. Indeed, this question is at the heart of understanding the dynamics of key microbial communities, including the human microbiome.

Defining bacterial and archaeal populations, and by extension species, is, therefore, an important endeavour for community ecology, but can we do it? Is microbial diversity organized into natural units to which we can ascribe biologically meaningful properties? Specifically, do fundamental evolutionary processes organize coexisting genotypes into units through which adaptations can specifically spread, giving rise to ecological units with clearly different dynamics? If we can define microbial populations in such a way, then we may be able to apply the rich evolutionary and ecological theory developed for animals and plant populations [7,8]; if not, then we might need fundamentally different theory and approaches [2].

Here, we explore the question of whether bacteria are organized into genetically clearly delineated, ecologically differentiated populations. We argue that although bacterial and archaeal recombination, both homologous and non-homologous, is unidirectional and promiscuous, environmental structure and selection have the potential to structure gene flow sufficiently for ecologically differentiated units to arise. We next discuss why recognizing such units has remained so difficult and show that, by estimating only very recent gene flow, congruent units of gene flow and ecology are indeed recovered. Although many more examples are still needed, these units may be the bacterial and archaeal equivalent of populations and their identification may ultimately contribute to solving the microbial species problem. We conclude by drawing implications for measuring biologically meaningful diversity in the environment.

## 2. Should we expect to find clearly delineated populations among bacteria and archaea?

**Figure 1.**
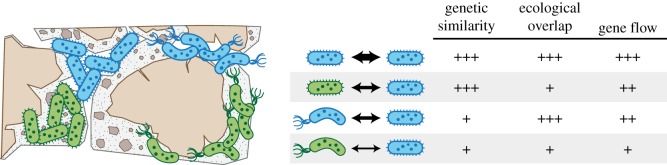
The magnitude of gene flow between microbial populations is shaped predominantly by the genetic similarity and ecological overlap of the individual strains that make up those populations. While the efficiency of homologous recombination decreases exponentially with sequence divergence, the likelihood of transfer increases with greater physical contact between strains that occupy similar physical niches. (Online version in colour.)

Although gene flow is potentially promiscuous in the sense that any microbe can, in principle, share genes with any other [9,10], it need only be structured enough to allow for preferential adaptations to spread in order for populations as local ecological units to emerge [11,12]. Consider that populations, which occupy a defined habitat, consist of individuals that are under similar selective pressures because they coexist and carry out similar functions (figure 1). Such habitats may be small organic particles in soils or aquatic environments, or more expansive bodies of water with defined physical and chemical properties [13–15]. However, the key is that habitats are nearly always patchy and ephemeral, and that they allow for a subset of populations within the community to increase in abundance through preferential growth [13,16–18]. As a result, active populations have a higher probability of sharing genetic material because homologous recombination rates decrease exponentially with sequence divergence [19,20] and preferential microhabitat associations ensure higher encounter rates (figure 1).

This increased encounter and recombination of actively growing genotypes has important consequences for creating and maintaining ecological cohesion [12]. If an adaptation arises within a population, it will spread more easily within the population, owing to the combination of preferential gene flow and fitness increase in the genotypes carrying the adaptation [11]. In other words, depending on the balance between the strength of selection and rate of recombination, the adaptation may spread through the population by a selective sweep [12,21]. If the adaptation is useful to other coexisting populations, its fitness advantage to a particular population is short-lived because horizontal gene transfer probably makes it available to other populations [22]. However, the scenario can be quite different if trade-offs are associated with the carriage of the adaptation, meaning that it may not function as well in a different genomic or ecological background [12,23,24]. If this is the case, an adaptation may remain population- or species-specific for much longer and enforce ecological differentiation. Trade-offs can also initiate the process of speciation if genotypes carrying the adaptation are more fit in a new habitat but less so in the ancestral habitat [12,23]. This effect may induce physical separation and thus a gene flow barrier between the nascent populations [12,25].

The trade-offs discussed above are often difficult to identify because they require the examination of very recently speciated populations. Among more divergent species, too many genetic changes have typically accumulated as well as been lost to identify the trait associated with the trade-off. One clear example comes from recently speciated bacterial populations in the ocean [26]. A comparative genomic approach identified two populations of *Vibrio cyclitrophicus* that were differentially distributed in ocean samples, one being associated with organic particles and the other occurring free-living. Both populations contained genome regions that differentiated them, including regions that contained much reduced nucleotide diversity, indicating a recent sweep of a specific allele, as well as regions that showed differential gene presence as expected from recent population-specific additions or losses. Some of these differentiating alleles and genes were clearly associated with biofilm formation and attachment, leading to the hypothesis that the ability to associate with particles was either lost or gained in one of the populations [26].

This hypothesis of differential adaptation based on observed genetic differences was subsequently confirmed by behavioural observations of representatives of the two populations that suggested a competition–dispersal trade-off [27]. Microfluidics was used to create an ecological landscape resembling conditions in the ocean where small particles represent a habitat to which bacteria can attach and degrade the solid organic material [13,16]. This degradation process itself creates an ephemeral habitat of patches of dissolved organic material because the attached bacteria extracellularly degrade organic polymers faster than they can import the breakdown products into the cell [16]. A cloud of mono- or oligomers forms around the particle by diffusion, and this material can be consumed by motile bacteria [28]. When such conditions were simulated in the microfluidic system, the two populations appeared differentially adapted to the solid and dissolved resources, respectively. While one responded by attaching to the particles and growing in biofilms, the other was capable of efficient dispersal among particles, rapidly detecting them and swimming towards new particles [27]. This suggests that the latter population is indeed better adapted to the exploitation of ephemeral, soluble nutrient patches, while the first commits to the degradation of the solid organic material. Although difficult to prove, it was inferred from the genomic comparison that these behavioural differences were involved in the speciation process because the differential adaptations represent an ecological trade-off that cannot easily coexist in genomes.

Although the above example demonstrates the power of population genomics combined with fine-scale environmental sampling, the discovery of such recently speciated populations was nonetheless fortuitous. It was aided by the fact that a protein-coding gene used as a marker to differentiate isolates initially was linked to a sweep region and thus clearly differentiated these two populations [26]. In most cases, population structure cannot be inferred *a priori* and instead such inference requires an approach where some measure of diversity is mapped onto environmental samples. We next outline reasons for this difficulty of recognizing population or species boundaries among bacteria and archaea based on genetic information alone.

## 3. Why is it so difficult to define populations?

In a recent opinion piece, Rocha [2] outlined challenges in bacterial (and archaeal) population genetics in the light of the neutral theory of evolution. One of the most important problems is that it has been nearly impossible to define the object of the study because of its fuzzy nature. Similar arguments have been made earlier for species boundaries [29]. Such fuzziness is observed in phylogenetic trees of multiple loci across the genome because they result in different topologies. That is, although clustering is observed, it is inconsistent when different genes are considered, reflecting their divergent evolutionary history [29,30]. A recent paper even argued that recombination has been so promiscuous among *Escherichia coli* isolates that there is no majority tree, even though, paradoxically, a similar tree is always produced when averaging over different larger genome regions [31]. This is potentially problematic when, as in many recombination estimation methods, individual genes are compared to such a consensus tree that is supposed to reflect the clonal history (or clonal frame) of the population. Overall, these observations suggest that phylogenetic methods can encounter problems in delineating populations and species.

The issue with phylogenetic methods may be that they integrate over too long evolutionary timeframes to be useful for population differentiation. In particular, among recently speciated populations, only a very small fraction of the genome supports a distinction between them [26]. This is illustrated well in the analysis of two recently speciated *V. cyclitrophicus* populations, where essentially every genomic region they shared had its own unique evolutionary history and both populations appeared completely intermixed [26]. This is an apparent paradox: how can there be recombination across population boundaries while population-specific sweeps are observed? The answer lies in the time scales over which phylogenetic comparisons integrate. When a method was devised to analyse only the most recent recombination events, these were more frequent within populations. This suggests that while the two populations shared a common history of recombination, the most recent post-population-divergence recombination events were population-specific [26].

Even many methods designed to measure recombination may suffer from a similar problem of integrating over evolutionary timeframes that are too long to capture speciation events. We recently carried out a simple experiment where we simulated a burst of recombination among a group of otherwise clonally evolving genomes and observed how the signal of recombination decayed as mutations accumulated [32]. When recombination was analysed with two different methods that rely on the identification of homoplasies, there was still considerable signal long after gene flow was terminated. This is because homoplasies are only slowly erased by the random mutational process, so that methods relying on their measurement integrate over long periods of time and do not capture only the contemporary recombination process. Such integration over long timeframes becomes problematic when closely related populations or even species are being compared and suggests that methods capable of analysing more contemporary gene flow are needed to correctly recover population or species boundaries [32].

## 4. Can we estimate gene flow in the context of contemporary population structure?

**Figure 2.**
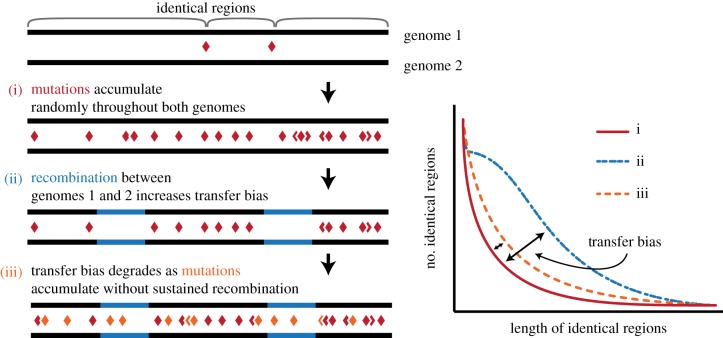
The method ‘populations as clusters of gene transfer’ (PopCOGenT) estimates the amount of recent horizontal gene transfer by measuring the distribution of lengths of identical sequences shared by any two genomes. By comparing this distribution to a null model of clonal evolution (i), PopCOGenT determines a ‘transfer bias’ owing to horizontal gene transfer. After the cessation of horizontal transfer between genomes, this transfer bias decays rapidly owing to the accumulation of mutations. (Online version in colour.)

If current methods cannot recover species or population boundaries, is there an alternative that can correctly identify such boundaries? We have recently proposed such a method that relies on measuring the homogenizing force of recombination between two genomes and is able to identify much more recent gene transfer than other methods [32]. This method, called populations as clusters of gene transfer (PopCOGenT), differs from others, in that it estimates recent gene transfer via shared identical genome regions (figure 2). Because such identical tracks between two closely related genomes can originate via vertical inheritance or horizontal gene transfer, PopCOGenT differentiates the two using a simple model of vertical (clonal) inheritance. If two genomes diverge clonally by mutational accumulation without recombination, they will have a characteristic length and frequency distribution of identical regions that can be estimated by a Poisson model of single-nucleotide polymorphisms [32]. Significant enrichment in identical regions above that expectation can then serve as an estimate of gene transfer (figure 2). The gene transfer signal decays by an order of magnitude within the time it takes for genomes to diverge by 0.1%, and PopCOGenT can, therefore, provide a much more contemporary measure of gene transfer than other methods [32].

**Figure 3.**
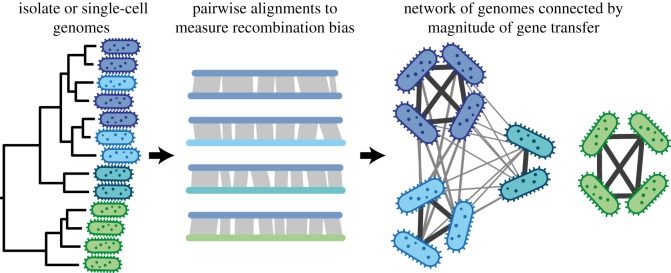
PopCOGenT identifies populations through pairwise whole-genome alignments of environmentally derived isolate or single-cell genomes. It is often unclear how to group strains together into biologically meaningful populations from phylogenetic trees made from multiple genome alignments or concatenated marker genes (left). Further, the diversity in these phylogenetic trees can only ever depict the evolutionary history of core genomic regions. By performing pairwise alignments, PopCOGenT estimates gene transfer across all regions shared by any two genomes and identifies population structure without relying on rigid identity cut-offs (middle). While some populations are completely disconnected from other groups by gene flow, others remain interconnected, and the underlying population structure is revealed through clustering that identifies subclusters of extensively connected strains (right). The isolated clusters of genomes can be considered species-like owing to the properties they share with the biological species concept definition requirement of genetic isolation. (Online version in colour.)

Importantly, the measure of gene transfer provided by PopCOGenT can be used to construct a network to examine how recombination structures genetic diversity (figure 3). In the example shown in figure 3, the individual genomes show different amounts of gene flow between them. Some isolates form a clearly isolated cluster, while others remain connected by considerable gene flow, yet are further structured into more weakly connected subclusters. As detailed below, such subclusters can be observed by applying a simple clustering algorithm to the raw gene flow network. Moreover, because PopCOGenT works with pairwise alignments, it can compare all shared regions, irrespective of whether these are shared by all isolates across a population. In that way, recently shared genetic material in both the core and flexible genome can be taken into account, i.e. in the gene complement that is shared by all and or subsets of isolates in a population, respectively.

When applied to several bacterial and archaeal model systems for which population structure has been estimated (using population genomics combined with ecological and physiological data), PopCOGenT was able to recapitulate the original predictions [32]. These model systems represent a critical test, as each has been shown to comprise closely related sister populations distinguished by cohesive properties, including differential dynamics in environmental samples. When PopCOGenT was used to construct a gene flow network among genomes from these model systems, the raw network was structured into gene flow clusters that were highly congruent with the previously identified genetic and ecological units.

These initial clusters in the raw gene flow network had no connection to other such clusters, indicating that recent gene flow between many ecological populations is essentially undetectable [32]. When a simple clustering algorithm was applied, however, the additional structure was revealed in some cases, i.e. subclusters of enriched gene flow within that maintain some gene flow between. These subclusters also recapitulated two models of recently diverged populations in *V. cyclitrophicus* and *Sulfulobus icelandicus* [26,33], indicating that PopCOGenT can correctly identify nascent populations separated by weaker gene flow discontinuities [32]. One of the datasets also consisted primarily of genomes amplified from single cells of the ocean cyanobacterium *Prochlorococcus*. Such single-cell genomes are usually difficult to compare by traditional methods because they are incomplete in random areas. However, PopCOGenT can handle incomplete information because it relies on pairwise comparisons as long as sufficient overlap between pairs is available. What constitutes sufficient information remains poorly explored and datasets can also easily be confounded by contaminating DNA that may be scored as gene transfer connections among unrelated genomes. Nonetheless, the potential to carry out population genomics with single-cell genomes and thus sidestep cultivation represents a potential advantage of PopCOGenT. Overall, the observation of clusters and subclusters among closely related genomes suggests that estimates of gene flow alone can be used to hypothesize genetic and ecological units. However, how can we be sure that the correct boundaries among these units have been identified?

## 5. How can we test if predicted population structure is biologically meaningful?

**Figure 4.**
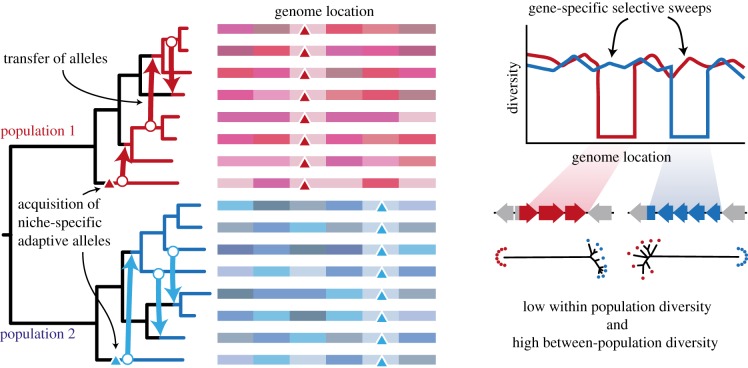
A major function of populations and species identified by gene flow is that they are the fundamental units through which adaptive traits radiate and spread. When alleles are acquired by a population (either through de novo mutation or horizontal acquisition from a distant relative), those alleles can be transferred to other members of the same population by homologous recombination. Further, if those traits provide a niche-specific benefit that substantially increases the fitness of their host, they will rise to fixation in that population owing to selection. Consequently, a hallmark of these regions when comparing genomes is locally diminished nucleotide diversity at the selected locus. The observation of these regions that have undergone recent selective sweeps are a useful confirmation that the predicted population structure is biologically meaningful. Indeed, randomized population groupings consistently prevent the identification of swept regions.

To answer this question, we return to the argument that for genetic and ecological units to be congruent, adaptations must be able to spread in a species- or population-specific manner. A critical test is, therefore, whether there are properties that differentiate the most closely related sister populations. Both examples of the speciation models of *V. cyclitrophicus* and *S. islandicus* suggest that such properties can be identified [26,33]. We, therefore, extended the logic of the gene flow analysis to the identification of alleles and genes that have swept in a population-specific manner [32] (figure 4). We reanalysed *Ruminococcus gnavus* genomes isolated from healthy individuals as well as patients with Crohn's disease and ulcerative colitis [34]. The application of PoCOGenT showed a connected network with three subclusters, two of which were sampled enough to test for adaptations in the form of population-specific alleles or genes [32]. For these adaptations to have arisen recently by population-specific sweeps, they should show much reduced diversity in the alleles or genes encoding them compared to the average nucleotide diversity across the genomes of the populations.

When a pipeline was developed to identify genome regions with significantly reduced nucleotide diversity compared to the population average (figure 4), several alleles in the core genome and genes in the flexible genome were identified that differentiated both populations [32]. These regions were all unlinked and distributed across the genome, indicating that they arose independently from each other. Many of these alleles and genes could not be annotated, but several encoded surface proteins, suggesting that they are involved in some form of communication with the environment. These results, therefore, suggest that gene flow is sufficiently biased in a population-specific manner to allow for adaptations to spread by recombination and serves as a strong confirmation that correct ecological units have been identified.

## 6. How can population structure evolve under horizontal gene transfer?

How can the observation of clearly delineated clusters in contemporary gene flow be reconciled with observations of horizontal gene transfer that has, in some cases, been called ‘rampant’ [35]? There is abundant evidence that there is a continuous uptake and incorporation of divergent genetic material into bacterial and archaeal genomes [25]. That is, each cell might at any point harbour genes that have recently been acquired from any number of other microbes. Although such incorporation of divergent genes will affect phylogenetic clustering of isolates, it will not disrupt the gene flow network sufficiently to mask population structure if the gene flow within populations is much higher than between, as we suggest here. Moreover, if the gene flow is fairly random, it will link strains between populations in a more or less haphazard way, so that connections are fairly unstructured. Indeed, many of the acquired genes may be lost fairly quickly if they are, as seems likely, at least slightly deleterious to the recipient genome [11]. Hence, populations and possibly species are indeed fuzzy units owing to horizontal gene transfer, but such fuzziness does not preclude their definition as ecological units if gene flow is sufficiently biased towards within-population recombination to allow for adaptations to sweep in a specific manner.

A constant sampling of genetic material from divergent sources can, in fact, provide the raw material for adaptation [11]. Although it is widely accepted that evolutionary innovation can arise by horizontal addition of genes into the genome, the extent to which even allelic sweeps arose horizontally rather than by mutation within the population was surprising in our recent analysis of the recently differentiated *Ruminococcus* populations discussed above. The vast majority of adaptive alleles we were able to identify were horizontally acquired from divergent sources [32]. Similarly, an adaptive radiation that differentiated closely related populations of ocean bacteria for different physical forms of the same polysaccharide was based on acquisition and loss dynamics of genes [36]. Even multiple copies of the same polysaccharide lyases originated by transfer rather than duplication, including some enzymes that were present in as many as seven copies per genome. These observations are consistent with previous analysis of diverse genomes that also showed duplication of genes within the same genome to be rare in microbes [37]. This is a fundamental difference to eukaryotes, where duplications are common and evolutionary innovation arises by mutation within the genome [38].

## 7. What are potential caveats of population structure predictions?

Considering that the results so far demonstrate the existence of surprisingly highly isolated gene flow clusters, are there potential scenarios where the horizontal transfer can mask or erase population structure? This aspect remains poorly explored, but several scenarios can at least be imagined. Recombination rates among microbes are highly variable [32,39], and if very low, the input of a larger set of genes from one other population may create a strong link with a subset of genomes in the population under consideration, confounding population structure analysis. The most likely scenario is a population with low recombination rates that receives a large mobile genetic element (MGE) that is under positive selection in both the donor and recipient population and thus connects a large fraction of the genomes. Such a case might originate if, for example, an antibiotic resistance plasmid moves through a microbiome under strong antibiotic selection. It is thus advisable to test population structure with and without MGEs, or to include closely related genomes from samples that have not been subject to antibiotic treatment. Moreover, it is possible that two related populations suddenly occupy similar niches owing to some environmental change. Such alteration in co-occurrence may allow for increased gene flow, especially if under selection, and lead to despeciation as has been postulated for some *Campylobacter* species in animal microbiomes [40]. Although these types of situations may lead to population structure that is less clear than those identified in the model systems we analysed, the gene flow patterns are nonetheless biologically relevant and may lead to interesting hypotheses about the environmental selection.

We stress that any population structure prediction represents a hypothesis in itself and needs to be carefully analysed as it may be affected by sampling and other factors. However, we believe that if populations carry signatures of specific adaptations, such as gene-specific sweeps (figure 4), these serve as some of the strongest possible evidence that the predicted population represents an ecological unit and hence the most relevant unit for community ecology.

## 8. What are key properties of populations defined by gene flow?

One striking feature of the populations identified here is that they contain relatively low nucleotide diversity in their core genome, i.e. in the genes shared by all. The genomes of both bacteria and archaea analysed so far are typically more than 98% similar in the nucleotide sequence within populations, which is consistent with data obtained from a different approach for predicting population structure [41]. Such high similarity would also ensure that homologous recombination within populations remains efficient, as its rate decays exponentially with sequence divergence [19,20]. It should also be noted that these low values are quite consistent with nucleotide diversity within animal and plant species. For example, human genomes differ by at most 0.2% of nucleotide sites compared to the human reference human genome [42].

If the populations defined by gene flow are taken as local representatives of species, they are considerably more narrowly defined than those resulting from the comparison of average nucleotide identity (ANI), which has become the basis of a popular species definition [43,44]. When ANI is compared across diverse groups of genomes, there is typically a minimum observed at around 95% ANI, the presumed species boundary [44]. However, this boundary probably does not conform to population or species boundaries for reasons similar to those voiced above concerning population boundaries estimated with some recombination methods. Once gene flow decreases owing to speciation, the genetic similarity between the nascent species will decay because recombination no longer acts as a homogenizing force [25]. Yet, this decay is a slow process and for the signal of genetic similarity to reach a minimum will take considerable time [32]. Hence, the population or species boundary may lie within the 95% similarity value, and, importantly, recently speciated populations may not be recognizable because their genomes have not diverged enough, masking ecological or disease associations as recently demonstrated [26,32,45]. Hence, while appealing for their simplicity, it is questionable whether ANI minima can define biologically meaningful species boundaries.

A further important property of populations defined by gene flow is that the pan-genome remains of considerable size [46]. That is, in spite of genomes being very closely related across the shared genes, they display a considerable number of genes that are not shared. Many of these genes remain unannotated and their role for population biology is thus unclear. However, there are an increasing number of examples which show that the flexible genome might, at least in part, be under negative frequency-dependent selection, a form of selection where the fitness of a genotype decreases as it becomes more frequent in the population [46]. This effect may be especially strong for organismal interaction such as public good production and predation. For example, the production of siderophores by some genotypes has been shown to be accompanied by the evolution of cheaters that lack the production genes but retain the uptake genes [47,48]. Moreover, viral receptors and defence genes are frequently relegated to the flexible genome, indicating that they cannot rise to high abundance within populations as protection against specific viruses decimating the population [46,49,50]. Finally, there is also increasing evidence that such flexible genome regions can be preferentially shared within populations by homologous recombination of the flanking regions so that rather than being repeatedly acquired de novo, many flexible regions are part of a population's biology [46].

## 9. What are the implications for measurement of diversity in the environment?

The approach for hypothesizing population structure based on gene flow followed by testing of the hypothesis by the identification of population-specific sweeps allows for a reverse ecology approach that predicts ecological units from genomic information alone [32,51]. In this way, the approach can provide an unbiased framework for identifying important variables that drive diversification in microbial populations by highlighting alleles and genes under strong selection. This approach thus provides a unique lens to delineate microbial niche space that is agnostic to being able to accurately measure where strains fall along environmental gradients. Of course, direct insights into ecological differentiation based on any genomic approaches depend heavily on the accuracy of gene annotations, which is currently patchy at best. But a reverse ecology approach can also help formulate hypotheses for relevant genes that need to be further characterized by other approaches such as molecular genetics or structural analysis and may thus help build a more structured approach towards solving the omnipresent annotation problem.

Loci under selection are particularly useful for assessing the abundance of populations in environmental samples because their within-population diversity is exceptionally low, while the diversity between populations is much higher because evidence so far indicates that most loci arose by horizontal gene transfer from divergent sources [32]. These properties mean that sweep loci can be detected with very high accuracy in environmental samples, and their prevalence throughout the genome of recombinogenic organisms adds statistical power in assessing the abundance of populations in complex communities. Accordingly, shotgun metagenomes of DNA extracted from microbial communities provide a convenient way to quantitatively assess the abundance of multiple loci in multiple samples. However, this approach is of limited use if target populations are rare in their environment. Sweep loci could also be targets for high-resolution assays such as digital polymerase chain reaction allowing researchers to rapidly measure the abundance of populations in different samples if greater sensitivity is required. These regions could also be targets for fluorescence *in situ* hybridization probes to directly visualize how closely related populations are differently distributed in the environment. We envision that this will allow for more targeted testing of fine-scale environmental associations that far exceeds the efficiency of traditional forward ecological approaches, which often rely on mapping microbial groups onto coarse environmental variables and then using genomics to find potential differences [12].

How do populations defined by gene flow compare to the traditional measurement of microbial diversity by 16S rRNA gene sequencing often used to map microbial populations onto environmental samples? To answer this question, we use an example from our own work where we have delineated *Vibrionaceae* bacteria into coexisting populations in ocean water. We typically find around 20 or so coexisting populations that were originally defined by the fine-scale environmental sampling of isolates, sequencing of protein marker genes and application of mathematical modelling to link genetic diversity to environmental structure [52–55]. These population predictions have recently been confirmed by the much simpler gene flow analysis [32] enabling direct comparison of one of the protein marker genes (*hsp60*) with different 16S rRNA gene fragments used to define operational taxonomic units (OTUs) for their potential to differentiate ecological units in samples.

**Figure 5.**
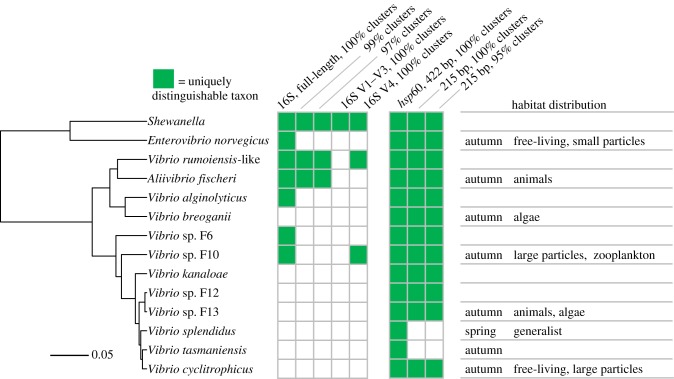
16S rRNA gene sequence clusters can distinguish 0–7 of 14 ecologically distinct *Vibrionaceae* populations depending on sequence length and clustering cut-off, while clusters in the *hsp60* marker gene can distinguish all or nearly all. Phylogeny is based on 52 concatenated ribosomal proteins. A shaded box indicates that a taxon can be uniquely distinguished with the given gene length and clustering method, while a white box indicates that a taxon is merged with at least one other taxon, in at least one gene cluster. Habitat distribution descriptions are derived from a quantitative analysis of populations' distributions across three different sample sets by Preheim *et al*. [54]. Taxa without habitat descriptions were excluded from that analysis because of limited sampling. (Online version in colour.)

This comparison shows disturbingly low resolution of the 16S rRNA genes when compared with populations defined by gene flow (figure 5). Especially 16S rRNA tags typically used in high throughput sequencing have essentially zero resolution for ecological populations. For the full-length gene, this is only slightly better, showing that speciation by far outpaces the resolution of the 16S rRNA genes. This means that the gene has very limited information when it comes to ecological dynamics of populations in environmental samples, and a unique sequence may mask many ecologically differentiated populations, an effect that obviously becomes worse the more broadly OTUs are defined in terms of sequence divergence.

Considering that the prediction of population structure by gene flow requires isolates or single-cell genomes, is there a proxy that can be developed for species and population identification in metagenomes? Potentially yes. One interesting feature of the populations we have identified is that they are quite well approximated by nearly identical ribosomal protein sequences [32,45]. Although even for these, some structure can be masked because of rapid speciation, these genes can serve nonetheless as a much more accurate proxy for population structure. Whether this observation holds more broadly across many taxa will have to be explored in larger datasets [56], but importantly, identical ribosomal proteins can be extracted from metagenomic datasets and their dynamics thus easily analysed [57]. We, therefore, recommend targeting ribosomal proteins when species and population dynamics are of interest in metagenomic samples.

## 10. Concluding remarks

The identification of populations as gene flow clusters that are also ecological units has major implications for microbiology, which has long suffered from the fuzzy definition of populations [2]. We suggest that recent gene flow measured from collections of closely related genomes can clearly delineate population boundaries even at relatively early stages of differentiation. These populations are characterized by alleles and genes that have recently swept to fixation, indicating that positive selection can spread adaptations in a specific and exclusive manner. The identification of such gene-specific sweeps provides both confidence in the population boundaries and creates hypotheses of recent adaptations that differentiate populations from each other. Hence, these populations can be regarded as adaptively optimized units of bacteria and archaea equivalent to how populations are viewed in macroecology and evolution. Such populations then hold significance when we want to study community ecology, as they allow for sharper identifications of associations with biotic and abiotic factors.

Finally, considering that many of the populations defined here display a very high degree of genetic isolation, it is tempting to invoke the biological species concept, which posits that species are reproductively isolated groups of organisms [58]. However, we stress that the analyses for bacteria and archaea presented here primarily considered organisms that either coexist or live in separate locations connected by high migration. As we outlined here, genetic isolation of such populations may be enforced by selection. Yet, a characteristic of many species is that they consist of geographically separate populations connected by various degrees of gene flow. How such structure influences the delineation of clusters remains an open question, but this will be important to determine in the pursuit of a biologically meaningful species concept for bacteria and archaea.

## Data accessibility

This article has no additional data.
